# 6-Chloro-2-(4-meth­oxy­phen­yl)-4-phenyl­quinoline

**DOI:** 10.1107/S1600536813023295

**Published:** 2013-08-23

**Authors:** B. Saravanan, V. Thamilarasan, N. Sengottuvelan, G. Chakkaravarthi, V. Manivannan

**Affiliations:** a Centre for Research and Development, PRIST University, Vallam, Thanjavur 613 403, India; b Department of Chemistry, DDE, Alagappa University, Karaikudi 630 003, India; c Department of Physics, CPCL Polytechnic College, Chennai 600 068, India

## Abstract

In the title compound, C_22_H_16_ClNO, the quinoline ring system makes dihedral angles of 56.30 (6) and 7.93 (6)°, respectively, with the adjacent phenyl and benzene rings. The dihedral angle between these phenyl and benzene rings is 56.97 (8)°. In the crystal, weak C—H⋯π and π–π [centroid–centroid distances of 3.7699 (9) and 3.8390 (9) Å] inter­actions link the mol­ecules into a layer parallel to the *ab* plane.

## Related literature

For standard bond lengths, see: Allen *et al.* (1987 ▶). For a related structure, see: Akkurt *et al.* (2004 ▶).
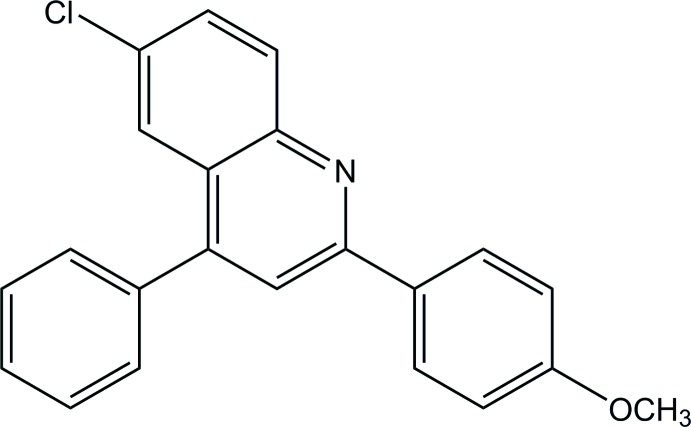


## Experimental

### 

#### Crystal data

C_22_H_16_ClNO
*M* *_r_* = 345.81
Monoclinic, 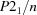

*a* = 10.5922 (5) Å
*b* = 8.2883 (3) Å
*c* = 19.1885 (9) Å
β = 92.988 (3)°
*V* = 1682.29 (13) Å^3^
*Z* = 4
Mo *K*α radiation
μ = 0.24 mm^−1^
*T* = 295 K
0.40 × 0.36 × 0.34 mm

#### Data collection

Bruker Kappa APEXII diffractometer
Absorption correction: multi-scan (*SADABS*; Sheldrick, 1996 ▶) *T*_min_ = 0.912, *T*_max_ = 0.924
12611 measured reflections
4148 independent reflections
3244 reflections with *I* > 2σ(*I*)
*R*_int_ = 0.034

#### Refinement

*R*[*F* ^2^ > 2σ(*F* ^2^)] = 0.043
*wR*(*F* ^2^) = 0.125
*S* = 1.03
4148 reflections
228 parameters
H-atom parameters constrained
Δρ_max_ = 0.29 e Å^−3^
Δρ_min_ = −0.44 e Å^−3^

### 

Data collection: *APEX2* (Bruker, 2004 ▶); cell refinement: *SAINT* (Bruker, 2004 ▶); data reduction: *SAINT*; program(s) used to solve structure: *SHELXS97* (Sheldrick, 2008 ▶); program(s) used to refine structure: *SHELXL97* (Sheldrick, 2008 ▶); molecular graphics: *PLATON* (Spek, 2009 ▶); software used to prepare material for publication: *SHELXL97*.

## Supplementary Material

Crystal structure: contains datablock(s) global, I. DOI: 10.1107/S1600536813023295/is5297sup1.cif

Structure factors: contains datablock(s) I. DOI: 10.1107/S1600536813023295/is5297Isup2.hkl

Supplementary material file. DOI: 10.1107/S1600536813023295/is5297Isup3.cml

Additional supplementary materials:  crystallographic information; 3D view; checkCIF report

## Figures and Tables

**Table 1:** Hydrogen-bond geometry (Å, °) *Cg*3 and *Cg*4 are the centroids of the C10–C15 and C16–C19 rings, respectively.

*D*—H⋯*A*	*D*—H	H⋯*A*	*D*⋯*A*	*D*—H⋯*A*
C14—H14⋯*Cg*4^i^	0.93	2.63	3.7695 (19)	151
C22—H22*B*⋯*Cg*3^ii^	0.96	2.84	3.613 (3)	138

Symmetry codes: (i) 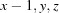; (ii) 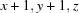.

**Fig. 1.**
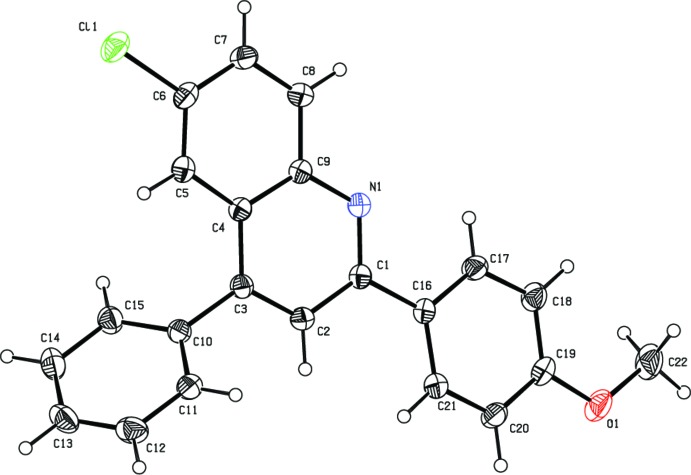
The molecular structure of the title compound, with atom labels and 30% probability displacement ellipsoids for non-H atoms.

## supplementary crystallographic information

### 1. Comment

The geometric parameters of the title compound (Fig. 1) are within the normal range (Allen *et al.*, 1987) and are comparable with the similar reported structure (Akkurt *et al.*, 2004). The quinoxaline ring system is almost planar [maximum deviation of C3 atom from the mean plane is 0.0345 (16)Å]. The dihedral angle between the phenyl ring (C10–C15) and methoxyphenyl ring (C16–C21) is 56.97 (8)°. The crystal structure exhibit weak C—H···π (Table 1) and π–π [*Cg*1···*Cg*2^i^ distance 3.7699 (9) Å and *Cg*2···*Cg*4^ii^ distance 3.8390 (9) Å; (i) -*x*, 1 - *y*, 2 - *z*; (ii) -*x*, 2 - *y*, 2 - *z*; *Cg*1, *Cg*2 and *Cg*4 are the centroids of the rings (C1/C2/C3/C4/C9/N1), (C4–C9) and (C16–C21), respectively] interactions which leads to the of packing of the molecules.

### 2. Experimental

5-Chloro-2-aminobenzophenone (1.86 g, 8.05 mmol) and 4-methoxyacetophenone (1.21 g, 8.05 mmol) in the presence of acetic acid (30 ml) and con. H_2_SO_4_ (0.5 ml) were stirred under argon at 140 °C for 18 h. After cooling to room temperature, 10% NaOH (100 ml) and dichloromethane (100 ml) were added to the reaction mixture. The organic layer was separated and washed with distilled water (50 ml×5) until a neutral solution was obtained. Later, it was dried over a Na_2_SO_4_ and evaporated under the natural condition to yield yellow crystals, suitable for X-Ray diffraction. Yield: 55 %.

### 3. Refinement

H atoms were positioned geometrically with C—H = 0.93 or 0.96 Å and allowed to ride on their parent atoms, with *U*_iso_(H) = 1.2*U*_eq_(C) or 1.5*U*_eq_(C_methyl_).

### Crystal data

**Table:**

C_22_H_16_ClNO	*F*(000) = 720
*M**_r_* = 345.81	*D*_x_ = 1.365 Mg m^−^^3^
Monoclinic, *P*2_1_/*n*	Mo *K*α radiation, λ = 0.71073 Å
Hall symbol: -P 2yn	Cell parameters from 5757 reflections
*a* = 10.5922 (5) Å	θ = 2.2–27.2°
*b* = 8.2883 (3) Å	µ = 0.24 mm^−^^1^
*c* = 19.1885 (9) Å	*T* = 295 K
β = 92.988 (3)°	Block, yellow
*V* = 1682.29 (13) Å^3^	0.40 × 0.36 × 0.34 mm
*Z* = 4	

### Data collection

**Table:**

Bruker Kappa APEXII diffractometer	4148 independent reflections
Radiation source: fine-focus sealed tube	3244 reflections with *I* > 2σ(*I*)
Graphite monochromator	*R*_int_ = 0.034
ω and φ scans	θ_max_ = 28.3°, θ_min_ = 2.7°
Absorption correction: multi-scan (*SADABS*; Sheldrick, 1996)	*h* = −12→14
*T*_min_ = 0.912, *T*_max_ = 0.924	*k* = −10→10
12611 measured reflections	*l* = −25→25

### Refinement

**Table:**

Refinement on *F*^2^	Secondary atom site location: difference Fourier map
Least-squares matrix: full	Hydrogen site location: inferred from neighbouring sites
*R*[*F*^2^ > 2σ(*F*^2^)] = 0.043	H-atom parameters constrained
*wR*(*F*^2^) = 0.125	*w* = 1/[σ^2^(*F*_o_^2^) + (0.0566*P*)^2^ + 0.6619*P*] where *P* = (*F*_o_^2^ + 2*F*_c_^2^)/3
*S* = 1.03	(Δ/σ)_max_ < 0.001
4148 reflections	Δρ_max_ = 0.29 e Å^−^^3^
228 parameters	Δρ_min_ = −0.44 e Å^−^^3^
0 restraints	Extinction correction: *SHELXL97* (Sheldrick, 2008), Fc^*^=kFc[1+0.001xFc^2^λ^3^/sin(2θ)]^-1/4^
Primary atom site location: structure-invariant direct methods	Extinction coefficient: 0.030 (2)

### Special details

**Table:**

Geometry. All esds (except the esd in the dihedral angle between two l.s. planes) are estimated using the full covariance matrix. The cell esds are taken into account individually in the estimation of esds in distances, angles and torsion angles; correlations between esds in cell parameters are only used when they are defined by crystal symmetry. An approximate (isotropic) treatment of cell esds is used for estimating esds involving l.s. planes.
Refinement. Refinement of F^2^ against ALL reflections. The weighted R-factor wR and goodness of fit S are based on F^2^, conventional R-factors R are based on F, with F set to zero for negative F^2^. The threshold expression of F^2^ > 2sigma(F^2^) is used only for calculating R-factors(gt) etc. and is not relevant to the choice of reflections for refinement. R-factors based on F^2^ are statistically about twice as large as those based on F, and R- factors based on ALL data will be even larger.

### Fractional atomic coordinates and isotropic or equivalent isotropic displacement parameters (Å^2^)

**Table:**

	*x*	*y*	*z*	*U*_iso_*/*U*_eq_	
C1	0.10650 (13)	0.86173 (17)	0.92160 (8)	0.0315 (3)	
C2	0.00131 (14)	0.82128 (18)	0.87652 (8)	0.0344 (3)	
H2	0.0020	0.8475	0.8294	0.041*	
C3	−0.10138 (14)	0.74421 (18)	0.90118 (8)	0.0325 (3)	
C4	−0.09729 (14)	0.69944 (17)	0.97278 (7)	0.0317 (3)	
C5	−0.19335 (15)	0.61042 (18)	1.00396 (8)	0.0364 (3)	
H5	−0.2641	0.5763	0.9772	0.044*	
C6	−0.18207 (15)	0.57495 (19)	1.07298 (9)	0.0384 (3)	
C7	−0.07731 (16)	0.6223 (2)	1.11478 (8)	0.0429 (4)	
H7	−0.0724	0.5979	1.1621	0.052*	
C8	0.01818 (16)	0.7050 (2)	1.08569 (8)	0.0418 (4)	
H8	0.0888	0.7354	1.1134	0.050*	
C9	0.01122 (14)	0.74491 (18)	1.01414 (7)	0.0334 (3)	
C10	−0.21428 (14)	0.71165 (18)	0.85403 (8)	0.0344 (3)	
C11	−0.20230 (16)	0.6298 (2)	0.79205 (8)	0.0417 (4)	
H11	−0.1237	0.5911	0.7805	0.050*	
C12	−0.30662 (19)	0.6052 (2)	0.74711 (9)	0.0527 (5)	
H12	−0.2981	0.5497	0.7055	0.063*	
C13	−0.42299 (18)	0.6626 (2)	0.76372 (10)	0.0523 (5)	
H13	−0.4929	0.6459	0.7333	0.063*	
C14	−0.43653 (17)	0.7446 (2)	0.82506 (10)	0.0495 (4)	
H14	−0.5154	0.7836	0.8361	0.059*	
C15	−0.33255 (16)	0.7688 (2)	0.87029 (9)	0.0420 (4)	
H15	−0.3417	0.8238	0.9120	0.050*	
C16	0.21525 (14)	0.95295 (17)	0.89617 (8)	0.0323 (3)	
C17	0.30751 (16)	1.0092 (2)	0.94365 (8)	0.0403 (4)	
H17	0.3017	0.9842	0.9906	0.048*	
C18	0.40815 (16)	1.1014 (2)	0.92371 (9)	0.0440 (4)	
H18	0.4691	1.1366	0.9569	0.053*	
C19	0.41774 (15)	1.14080 (19)	0.85434 (9)	0.0391 (4)	
C20	0.32825 (16)	1.0835 (2)	0.80568 (9)	0.0423 (4)	
H20	0.3349	1.1082	0.7587	0.051*	
C21	0.22887 (15)	0.9899 (2)	0.82607 (8)	0.0382 (3)	
H21	0.1701	0.9509	0.7925	0.046*	
C22	0.60395 (19)	1.2957 (3)	0.87780 (12)	0.0627 (6)	
H22A	0.5638	1.3594	0.9121	0.094*	
H22B	0.6622	1.3618	0.8539	0.094*	
H22C	0.6487	1.2079	0.9004	0.094*	
N1	0.11065 (12)	0.82580 (16)	0.98859 (6)	0.0354 (3)	
O1	0.51123 (12)	1.23407 (17)	0.82929 (7)	0.0548 (3)	
Cl1	−0.30154 (4)	0.46790 (6)	1.11096 (3)	0.05376 (17)	

### Atomic displacement parameters (Å^2^)

**Table:**

	*U*^11^	*U*^22^	*U*^33^	*U*^12^	*U*^13^	*U*^23^
C1	0.0295 (7)	0.0299 (7)	0.0352 (7)	0.0002 (6)	0.0024 (6)	−0.0002 (6)
C2	0.0343 (7)	0.0371 (8)	0.0318 (7)	−0.0013 (6)	0.0014 (6)	0.0031 (6)
C3	0.0314 (7)	0.0324 (7)	0.0337 (7)	−0.0014 (6)	0.0006 (6)	−0.0011 (6)
C4	0.0314 (7)	0.0308 (7)	0.0331 (7)	0.0001 (6)	0.0034 (6)	−0.0002 (6)
C5	0.0324 (7)	0.0357 (8)	0.0413 (8)	−0.0020 (6)	0.0037 (6)	−0.0004 (6)
C6	0.0377 (8)	0.0345 (8)	0.0442 (8)	0.0026 (6)	0.0133 (7)	0.0052 (6)
C7	0.0456 (9)	0.0502 (10)	0.0335 (8)	0.0033 (8)	0.0059 (7)	0.0064 (7)
C8	0.0396 (8)	0.0532 (10)	0.0325 (7)	−0.0024 (7)	−0.0003 (6)	0.0026 (7)
C9	0.0316 (7)	0.0351 (7)	0.0336 (7)	0.0008 (6)	0.0030 (6)	−0.0003 (6)
C10	0.0328 (8)	0.0359 (8)	0.0344 (7)	−0.0066 (6)	−0.0002 (6)	0.0045 (6)
C11	0.0393 (9)	0.0485 (9)	0.0374 (8)	−0.0043 (7)	0.0035 (7)	−0.0005 (7)
C12	0.0616 (12)	0.0567 (11)	0.0390 (9)	−0.0115 (9)	−0.0044 (8)	−0.0038 (8)
C13	0.0484 (10)	0.0555 (11)	0.0511 (10)	−0.0127 (9)	−0.0169 (8)	0.0077 (8)
C14	0.0343 (9)	0.0498 (10)	0.0638 (11)	−0.0017 (7)	−0.0044 (8)	0.0068 (9)
C15	0.0375 (8)	0.0448 (9)	0.0435 (9)	−0.0014 (7)	0.0001 (7)	−0.0024 (7)
C16	0.0284 (7)	0.0319 (7)	0.0369 (7)	0.0004 (6)	0.0035 (6)	−0.0008 (6)
C17	0.0405 (9)	0.0459 (9)	0.0346 (7)	−0.0094 (7)	0.0039 (6)	0.0007 (7)
C18	0.0381 (8)	0.0482 (9)	0.0458 (9)	−0.0119 (7)	0.0043 (7)	−0.0055 (7)
C19	0.0349 (8)	0.0357 (8)	0.0481 (9)	−0.0024 (6)	0.0139 (7)	−0.0004 (7)
C20	0.0415 (9)	0.0478 (9)	0.0386 (8)	−0.0002 (7)	0.0111 (7)	0.0035 (7)
C21	0.0339 (8)	0.0437 (9)	0.0368 (8)	−0.0016 (6)	0.0012 (6)	−0.0002 (6)
C22	0.0443 (10)	0.0639 (13)	0.0804 (14)	−0.0197 (9)	0.0077 (10)	0.0101 (11)
N1	0.0310 (6)	0.0405 (7)	0.0345 (6)	−0.0028 (5)	0.0010 (5)	0.0012 (5)
O1	0.0450 (7)	0.0609 (8)	0.0599 (8)	−0.0171 (6)	0.0170 (6)	0.0020 (6)
Cl1	0.0495 (3)	0.0526 (3)	0.0610 (3)	−0.00404 (19)	0.0196 (2)	0.0144 (2)

### Geometric parameters (Å, º)

**Table:**

C1—N1	1.3180 (19)	C12—C13	1.374 (3)
C1—C2	1.415 (2)	C12—H12	0.9300
C1—C16	1.482 (2)	C13—C14	1.373 (3)
C2—C3	1.367 (2)	C13—H13	0.9300
C2—H2	0.9300	C14—C15	1.381 (2)
C3—C4	1.422 (2)	C14—H14	0.9300
C3—C10	1.486 (2)	C15—H15	0.9300
C4—C9	1.413 (2)	C16—C17	1.382 (2)
C4—C5	1.415 (2)	C16—C21	1.394 (2)
C5—C6	1.356 (2)	C17—C18	1.382 (2)
C5—H5	0.9300	C17—H17	0.9300
C6—C7	1.391 (2)	C18—C19	1.379 (2)
C6—Cl1	1.7366 (16)	C18—H18	0.9300
C7—C8	1.365 (2)	C19—O1	1.3635 (18)
C7—H7	0.9300	C19—C20	1.380 (2)
C8—C9	1.410 (2)	C20—C21	1.381 (2)
C8—H8	0.9300	C20—H20	0.9300
C9—N1	1.3612 (19)	C21—H21	0.9300
C10—C11	1.381 (2)	C22—O1	1.413 (2)
C10—C15	1.390 (2)	C22—H22A	0.9600
C11—C12	1.381 (2)	C22—H22B	0.9600
C11—H11	0.9300	C22—H22C	0.9600
			
N1—C1—C2	121.87 (13)	C14—C13—C12	120.35 (16)
N1—C1—C16	116.71 (13)	C14—C13—H13	119.8
C2—C1—C16	121.37 (13)	C12—C13—H13	119.8
C3—C2—C1	120.97 (13)	C13—C14—C15	119.68 (17)
C3—C2—H2	119.5	C13—C14—H14	120.2
C1—C2—H2	119.5	C15—C14—H14	120.2
C2—C3—C4	118.15 (13)	C14—C15—C10	120.54 (16)
C2—C3—C10	120.24 (13)	C14—C15—H15	119.7
C4—C3—C10	121.60 (13)	C10—C15—H15	119.7
C9—C4—C5	118.91 (13)	C17—C16—C21	117.17 (14)
C9—C4—C3	117.13 (13)	C17—C16—C1	119.35 (14)
C5—C4—C3	123.95 (14)	C21—C16—C1	123.45 (14)
C6—C5—C4	119.84 (15)	C18—C17—C16	122.22 (15)
C6—C5—H5	120.1	C18—C17—H17	118.9
C4—C5—H5	120.1	C16—C17—H17	118.9
C5—C6—C7	121.92 (15)	C19—C18—C17	119.66 (15)
C5—C6—Cl1	119.45 (13)	C19—C18—H18	120.2
C7—C6—Cl1	118.63 (13)	C17—C18—H18	120.2
C8—C7—C6	119.46 (15)	O1—C19—C18	124.46 (16)
C8—C7—H7	120.3	O1—C19—C20	116.26 (15)
C6—C7—H7	120.3	C18—C19—C20	119.28 (14)
C7—C8—C9	120.90 (15)	C19—C20—C21	120.57 (15)
C7—C8—H8	119.6	C19—C20—H20	119.7
C9—C8—H8	119.6	C21—C20—H20	119.7
N1—C9—C8	117.64 (14)	C20—C21—C16	121.04 (15)
N1—C9—C4	123.43 (13)	C20—C21—H21	119.5
C8—C9—C4	118.93 (14)	C16—C21—H21	119.5
C11—C10—C15	119.02 (15)	O1—C22—H22A	109.5
C11—C10—C3	120.43 (14)	O1—C22—H22B	109.5
C15—C10—C3	120.50 (14)	H22A—C22—H22B	109.5
C10—C11—C12	120.26 (16)	O1—C22—H22C	109.5
C10—C11—H11	119.9	H22A—C22—H22C	109.5
C12—C11—H11	119.9	H22B—C22—H22C	109.5
C13—C12—C11	120.15 (17)	C1—N1—C9	118.39 (13)
C13—C12—H12	119.9	C19—O1—C22	117.74 (14)
C11—C12—H12	119.9		
			
N1—C1—C2—C3	0.7 (2)	C10—C11—C12—C13	0.2 (3)
C16—C1—C2—C3	−176.68 (14)	C11—C12—C13—C14	−0.1 (3)
C1—C2—C3—C4	−2.7 (2)	C12—C13—C14—C15	−0.2 (3)
C1—C2—C3—C10	176.23 (14)	C13—C14—C15—C10	0.3 (3)
C2—C3—C4—C9	2.8 (2)	C11—C10—C15—C14	−0.2 (2)
C10—C3—C4—C9	−176.10 (14)	C3—C10—C15—C14	177.21 (15)
C2—C3—C4—C5	−175.86 (14)	N1—C1—C16—C17	−6.4 (2)
C10—C3—C4—C5	5.2 (2)	C2—C1—C16—C17	171.08 (15)
C9—C4—C5—C6	2.2 (2)	N1—C1—C16—C21	175.34 (14)
C3—C4—C5—C6	−179.14 (15)	C2—C1—C16—C21	−7.2 (2)
C4—C5—C6—C7	−0.6 (2)	C21—C16—C17—C18	1.4 (2)
C4—C5—C6—Cl1	179.16 (11)	C1—C16—C17—C18	−176.93 (15)
C5—C6—C7—C8	−1.0 (3)	C16—C17—C18—C19	0.6 (3)
Cl1—C6—C7—C8	179.27 (13)	C17—C18—C19—O1	178.35 (16)
C6—C7—C8—C9	0.9 (3)	C17—C18—C19—C20	−1.8 (3)
C7—C8—C9—N1	−179.24 (15)	O1—C19—C20—C21	−179.09 (15)
C7—C8—C9—C4	0.7 (2)	C18—C19—C20—C21	1.0 (3)
C5—C4—C9—N1	177.71 (14)	C19—C20—C21—C16	1.0 (3)
C3—C4—C9—N1	−1.0 (2)	C17—C16—C21—C20	−2.2 (2)
C5—C4—C9—C8	−2.2 (2)	C1—C16—C21—C20	176.10 (14)
C3—C4—C9—C8	179.04 (14)	C2—C1—N1—C9	1.2 (2)
C2—C3—C10—C11	54.3 (2)	C16—C1—N1—C9	178.68 (12)
C4—C3—C10—C11	−126.74 (17)	C8—C9—N1—C1	178.93 (14)
C2—C3—C10—C15	−123.02 (17)	C4—C9—N1—C1	−1.0 (2)
C4—C3—C10—C15	55.9 (2)	C18—C19—O1—C22	−0.6 (3)
C15—C10—C11—C12	−0.1 (2)	C20—C19—O1—C22	179.54 (17)
C3—C10—C11—C12	−177.51 (15)		

### Hydrogen-bond geometry (Å, º)

*Cg*3 and *Cg*4 are the centroids of the C10–C15 and C16–C19 rings, respectively.

**Table:**

*D*—H···*A*	*D*—H	H···*A*	*D*···*A*	*D*—H···*A*
C14—H14···*Cg*4^i^	0.93	2.63	3.7695 (19)	151
C22—H22*B*···*Cg*3^ii^	0.96	2.84	3.613 (3)	138

Symmetry codes: (i) *x*−1, *y*, *z*; (ii) *x*+1, *y*+1, *z*.
